# Inferring Sexually Transmitted Infection Risk From Attractiveness in Online Dating Among Adolescents and Young Adults: Exploratory Study

**DOI:** 10.2196/14242

**Published:** 2020-06-09

**Authors:** Tamar Krishnamurti, Alexander L Davis, Baruch Fischhoff

**Affiliations:** 1 Division of General Internal Medicine University of Pittsburgh Pittsburgh, PA United States; 2 Engineering and Public Policy Carnegie Mellon University Pittsburgh, PA United States; 3 Institute for Politics and Strategy Carnegie Mellon University Pittsburgh, PA United States

**Keywords:** risk perception, heuristics, sexually transmitted infections, online dating, dating apps, adolescents, sexual health, attractiveness, halo effect

## Abstract

**Background:**

Sexually transmitted infection (STI) rates are on the rise among adolescents and young adults in the United States. With the popularity of online dating, adolescents and young adults must increasingly rely on limited cues to make initial judgments about potential sexual partners, including judgments about STI risk.

**Objective:**

This study aimed to assess whether in the context of online dating, an attractiveness heuristic would be used for STI risk assessment. We hypothesized that consistent with research on halo effects, decision makers would judge more attractive people to be less likely to have STIs.

**Methods:**

In a survey experiment, we asked participants to determine which individual in each of 20 sets of paired photographs was enrolled in a personals website for people with publicly disclosed STIs.

**Results:**

Despite financial incentives for accuracy and high levels of self-confidence in their judgments, participants performed no better than chance at identifying individuals with self-reported STIs. Contrary to our hypothesis, however, more attractive people were judged as being more likely to have an STI. This relationship appears to be mediated by inferences regarding the target individual’s sexual behavior, with more attractive individuals considered to have more partners.

**Conclusions:**

On showing adolescents and young adults photographs offering no diagnostic information about STIs, they appeared to use attractiveness as a cue for sexual risk, which was mediated by the belief that attractive individuals have more sexual opportunities. Health care providers may wish to address this heuristic process among their adolescent patients in discussions about sexual health.

## Introduction

In the United States, sexually transmitted infections (STIs) are increasingly among the most commonly reported diseases, with the total cases of STIs reaching a historical high in 2017 [1,2]. Young adults and adolescents are at particular risk [2]. Public health officials have suggested that online dating and use of dating apps may play critical roles in this burgeoning problem. Over half of the users of popular dating apps are in the highest STI age bracket (under 25 years) [3]. Some young adults have a belief that they can “just tell” by looking whether a potential partner has an STI [4,5]. In a recent study, among a sample of young adults using dating apps, those who transitioned from a profile view to having an in-person date had higher self-reported rates of risky sexual behavior than those who did not transition to face-to-face interactions [6]. Here, we examine one aspect of the process that can lead to sexual contact and STI risk, which is the inference that young people make regarding potential partners’ STI risk according to their personal appearance.

The interfaces of popular dating sites, such as Tinder, feature photographs of potential partners and encourage scrolling quickly through them and swiping “right” on appealing individuals for further examination [7]. Beneath the photographs are profiles that might be consulted for those who pass such initial screening. As it is socially awkward to ask about STI status [8] and dating apps rarely provide this information [9], young people must rely on intuition when making judgments about potential partners’ STI risk. Attractiveness can be a valid cue for predicting disfiguring STIs (eg, advanced syphilis). On the other hand, for the far more frequent cases of asymptomatic STIs, attractiveness provides no directly relevant information. However, attractiveness could provide indirectly relevant information if it is correlated with risk factors, such as number of sexual partners, frequency of sex, access to health care, and use of condoms.

Existing research offers conflicting evidence regarding the roles of attractiveness judgment in inferences regarding STI risk. Some studies found that when asked explicitly, young people expect more attractive individuals to have greater STI risk, reasoning that they will have more opportunities for varied sexual activities and be more promiscuous [10,11]. A large body of studies, however, suggested the opposite. For example, one study found that male respondents provided lower estimates of STI risk for women described as “attractive” in thumbnail personality sketches [12]. Another found an inverse relationship between how “attractive” various qualities were perceived in a potential partner and how “risky” those qualities were judged to be [13]. These studies may involve a “halo effect,” whereby one positive feature, such as physical attractiveness, encourages other positive perceptions [14,15]. Indeed, some evidence suggests that motivated rather than deductive reasoning may color subjects’ judgments about sexual purity, when self-justifying their failures to use condoms with more attractive partners [16,17]. There is also strong evidence that people seen as more attractive are also viewed as more intelligent, academically and socially competent, politically knowledgeable, and cooperative [18-23]. This halo effect is present for visual judgments of both male and female individuals and remains in place once individuals have interacted with one another. If sexual risk is within the halo of attractiveness, young people may infer a lower STI risk from potential partners’ physical attractiveness in online dating contexts.

In this study, we created such a context experimentally in order to examine whether attractiveness is a cue for risk when young people make judgments from photographic cues alone. We also examined their confidence and accuracy. We posited that people making quick judgments about potential dating partners in online dating profiles would apply an *attractiveness heuristic*. Namely, they would perceive attractive individuals as less likely to have an STI.

Previous studies of perceived STI risk have typically asked participants to make inferences from multiple (sometimes contradictory) cues [10,12] or have asked them to evaluate attractiveness and risk simultaneously, with explanation of their inferences [10-13,24]. Our study adds to this research by examining rapid judgments based on visual cues alone, with no prompt for reasoned inferences. Its results have implications for online dating contexts and, more generally, for the connections between fast and slow thinking [25].

We asked participants to judge the likelihood that individuals in photographs have STIs under conditions that should automatically evoke judgments of the targets’ attractiveness (making snap judgments based on rapidly displayed pairs of photographs). The judgment is which of the two pictures has been drawn from a website for people with self-disclosed STIs. To develop this test set of photographs, we had a separate sample rate the target in each photograph in terms of attractiveness and several factors that might mediate the relationship with STI risk (number of sexual partners [as a proxy for STI exposure], intelligence, frequency of condom use, and decision-making competence [potentially protecting from exposure]).

## Methods

### Baseline Photograph Data Set

To characterize the photographs used in the experiment, we had a separate pretest sample of 125 heterosexual individuals (aged 18-25 years; 39% female), who were recruited from a university student pool, rate opposite-sex photographs for attractiveness and several risk-related characteristics. Raters were recruited for a web-based survey in which they would be “asked to make judgments about individuals,” and they received university course credit for participating. They were eligible for the rating study if they self-identified as heterosexual, were at least 18 years of age, and self-reported not being in a romantic relationship. Photographs were drawn from profiles of residents across the United States. All photographs showed someone who identified as being between the ages of 18 and 25 years and heterosexual in their original online dating profile. Each individual was photographed looking directly at the camera. Among photographs of both male and female individuals, there were approximately 80% Caucasian people. Photographs were cropped square (1:1 aspect ratio) to show only the neck and face and to minimize surrounding visuals. Photographs were all in color and were selected by two independent research assistants as having a pleasant or neutral facial expression.

All photographs were publicly available, and use of the photographs complied with the terms of service of the websites at the time that the stimulus photographs were gathered. Although the photographs, by their nature, identified the individuals depicted, they were obtained from national sources; hence, there was a very low probability of including individuals known to the participants. The sources of the photographs were not revealed to the participants.

There were 96 photographs in total (48 photographs of male individuals and 48 of female individuals). Each photograph was rated by 10 raters. Each rater judged 12 unique, randomly selected, opposite-sex photographs. Photographs were rated on physical attractiveness (1 [very unattractive] to 7 [very attractive]), according to the approach in other studies on facial attractiveness [26,27]. Photographs were also rated on the following three risk-related characteristics drawn from prior studies [15], which could be protective against sexual risk: (1) *intelligence* (1 [not at all intelligent] to 7 [very intelligent]), (2) *competence* (1 [foolish] to 7 [sensible]), and (3) *condom use with a new partner* (1 [never] to 7 [always]). Additionally, photographs were rated on the following factor that could increase sexual risk: *likelihood of multiple sexual partners* (1 [not at all likely] to 7 [very likely]). Raters used the entire 7-point Likert scale. The average attractiveness score in ratings of photographs of male individuals ranged from 1.75 to 5.0 and in ratings of photographs of female individuals ranged from 1.35 to 5.50. Controlling for the website from which the photographs were drawn, photographs of male individuals were rated as slightly less attractive than those of female individuals (t_77_=2.11, *P*=.04). There were no significant differences in the attractiveness ratings of photographs from each dating website (*P*=.11; the average attractiveness ratings for STI website photographs ranged from 1.61 to 5.00 and for non-STI website photographs ranged from 1.35 to 5.50).

Descriptive statistics for the pretest sample’s ratings of the photographs that were ultimately retained to be used in the research study (10 pairs of male individuals and 10 pairs of female individuals; rated on a scale from 1 to 7) along with the correlations between them can be found in Multimedia Appendix 1. Individuals judged as more attractive were given higher ratings on the three protective factors (intelligence, frequency of condom use, and competent decision-making) and one risk factor (multiple sexual partners).

### Study Participants

Our study included 87 participants (55 male and 32 female participants) recruited from a private university student participant pool using online postings and recruited on the street in a high foot-traffic neighborhood housing multiple universities (both public and private) with a sign posted outside a research laboratory. The posting stated that participants were being recruited for “a study to understand individual decision-making” and could participate if they met the inclusion criteria of being at least 18 years of age and not participating in rating the baseline photograph data set. Participants recruited from the university student participant pool were emailed a link to the study. Participants recruited on the street outside the laboratory completed the study on a computer inside a private cubicle.

### Study Procedure

Participants were shown 20 pairs of photographs (10 pairs of male individuals and 10 pairs of female individuals) drawn from the prerated photograph set. One photograph in each pair was drawn from a personals website for people who have publicly disclosed an STI. The other photograph was drawn from a dating website without that disclosure. Participants were told about the two websites and the photograph sampling procedure. They were also asked to assume that people from the non-STI disclosure website had the same rate of STIs as the general population. For each photograph pair, participants were asked which photograph was from the STI website and about their confidence in the choice (from 50% [chance] to 100% [certainty]). For each pair, one photograph was randomly sampled from each site and assigned randomly to the right- or left-hand side.

Participants received US $5 in compensation for their time or university course credit. All participants received an additional US $0.25 for each correct response. This amount was selected to provide an incentive for accuracy without compromising the rapid judgment process. Such incentives have been found to increase attention without reducing errors attributable to heuristic use [28].

For those who completed the study online, the primary researcher evaluated responses for accuracy and emailed the participants about their payment, which was collected from another researcher. Although this researcher could infer the number of correct responses from the payment amount, there was no information about which stimuli a participant had seen. The data were fully deidentified upon payment and prior to the analysis.

After completing the task, participants answered questions about their relationship and sexual history, including binary response questions, such as *Are you currently in a romantic relationship?*, *Are you currently sexually active?*, and *Have you ever had a “one-night stand”?*, and numeric response questions, such as *How many sexual partners have you had in total?* and *How many times have you had sex in your lifetime?*, as well as a categorical sexual orientation question.

The study, including the acquisition and use of stimuli, was approved by the Institutional Review Board (IRB) of Carnegie Mellon University, which designated the study as posing minimal risk. The IRB did not require informed consent from the individuals in the stimuli as, at the time, all photographs were publicly available, with no requirement to create an account to view or download them for research purposes. To ensure the privacy of the individuals in the photographs, we have not made their images publicly available. Our code, survey, and stimuli rating data set are available publicly [29].

## Results

### Participant Characteristics

Of the 87 participants, 74 identified as heterosexual, 12 as homosexual or bisexual, and one did not respond. The participants ranged in age from 18 to 56 years, with a mean age of 22.8 (SD 7.8) years. Table 1 presents the participants’ self-reported demographic characteristics.

Among the 87 participants, 79 (91%) responded to the question about prior sexual activity, with 50% (26/52) of male participants and 22% (6/27) of female participants reporting none.

**Table 1:** Participants’ characteristics.

Variable		Value (N=87), n (%)
**Gender**		
	Male	55 (67%)
	Female	32 (33%)
**Age^a^**		
	18-24	65 (75%)
	25-34	10 (11%)
	≥35	5 (6%)
**Currently in a romantic relationship**		
	Yes	35 (40%)
	No	45 (52%)
**Currently sexually active**		
	Yes	42 (48%)
	No	38 (44%)
**Total lifetime instances of sex** (eg, penile-vaginal, oral, and anal)		
	0 instances	36 (41%)
	1-10 instances	13 (15%)
	>10 instances	31 (36%)
**History of “one-night stands”**		
	Never	67 (77%)
	At least once	13 (15%)

^a^Of the 87 participants, 7 (8%) did not provide complete demographic data.

### Descriptive Statistics

#### Sample Size Considerations

Each of the 87 participants made 20 judgments about which individual in a pair of photographs was more likely to have an STI. Our effective sample size is somewhere between 87 observations (if each participant’s responses are perfectly correlated) and 1740 observations (20 × 87). Assuming an effective sample size of 87, the statistical power is 0.15 to detect a small effect (*r*=0.1), 0.81 to detect a medium effect (*r*=0.3), and 0.99 to detect a large effect (*r*=0.5).

#### STI Identification

The mean percentage of correct STI identifications was 47% (9.85/20). Participants’ mean confidence in their judgments was 67.2% (SD 16.0%). Thus, in aggregate, participants were overconfident, expecting more correct identifications than were observed.

### Attractiveness Heuristic

To test for the use of an attractiveness heuristic in our primary experiment, we estimated a series of binary mixed logit models predicting the probability of participants predicting that an image was from the STI website as a function of the difference in the mean attractiveness ratings of the two images (for the pretest sample) and controlling for the “ground truth” of a self-disclosed STI. The following three models were created:


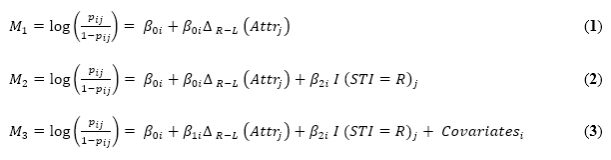


Models 1-3 in Table 2 are indexed by participant *i* and photograph pair *j.* The dependent variable *y_ij_* was coded as 1 if participant *i* selected the photograph on the right from photograph pair *j* and as 0 otherwise. Each model used the logit function to relate *p_ij_*, the modeled probability that participant *i* selects the right photograph in pair *j* as having an STI, to characteristics of the photograph and the participant. In model 1 (M_1_), we included an intercept *β_0i_* to allow for individual-specific tendencies to select the photograph on the right and *β_1i_* times the difference in attractiveness between the right and left photographs for pair *j* to capture individual-specific tendencies to use attractiveness as a cue for STI risk. The coefficients *β_0i_* and *β_1i_* are assumed to be drawn from a multivariate normal distribution with mean 0 and an unrestricted variance-covariance matrix. Model 2 (M_2_) adds an indicator variable for whether the photograph on the right involves an STI for photograph pair *j*, with a corresponding coefficient *β_2i_* that captures individual *i*’s ability to detect the presence of STI from the photographs. Model 3 (M_3_) adds additional individual-level covariates. For all models, we used the Nelder-Mead optimizer from the lme4 package in R for estimation.

As seen in Table 2, model 1 found that as the difference in attractiveness increases, participants are *more* likely to identify the more attractive individual as having been drawn from the STI website. This relationship held when, in model 2, the actual website was added to the equation, with that information not adding significant predictive power (consistent with participants predicting the actual website at chance level). Model 3 showed that the pattern held after adding four participant-level variables, none of which added predictive power (gender, age, gender match with the target individual, and the participant’s reported number of lifetime sexual partners).

Across all participants, a greater difference in attractiveness between the two photographs being judged was associated with a higher probability of the participant predicting that the more attractive individual was from the STI website (*Z*=2.08, *P*=.04).

**Table 2:** Mixed logit models predicting whether the target was drawn from the sexually transmitted infection disclosure website, according to attractiveness ratings, the actual website, and personal characteristics.

Variable	Model 1 (1738 observations, 87 participants)			Model 2 (1738 observations, 87 participants)				Model 3 (1578 observations, 79 participants)		
	Estimate	SE	SD		Estimate	SE	SD	Estimate	SE	SD
Intercept	−0.11	0.07	0.40		−0.08	0.08	0.41	−0.04	0.25	0.19
Attractiveness difference score	0.10^a^	0.05	0.20		0.10^a^	0.05	0.20	0.11^a^	0.05	0.21
Self-disclosed STI^b^	—^c^	—	—		−0.07	0.10	0.02	−0.07	0.10	0.07
Gender	—	—	—		—	—	—	0.12	0.11	—
Age	—	—	—		—	—	—	−0.001	0.01	—
Target matched on gender	—	—	—		—	—	—	0.05	0.10	—
Total number of sexual partners	—	—	—		—	—	—	0.02	0.05	—

^a^*P*<.05.

^b^STI: sexually transmitted infection.

^c^Not entered into the regression.

### Role of Risk-Relevant Characteristics in STI Risk Judgments

We used binary mixed logit models to assess the role of other features of the photographs, using pretest sample ratings (Multimedia Appendix 1) added to models 4-6 (Table 3). As seen in Table 3, model 4 found that the difference in target-perceived intelligence added no predictive power. Model 5 found that the difference in the ratings of condom use added predictive power, without affecting the relationship with attractiveness. Model 6 found that the difference in the ratings of the targets having multiple partners added predictive power as well, with attractiveness no longer playing a role.

**Table 3:** Mixed logit models predicting whether the target was drawn from the sexually transmitted infection disclosure website, according to attractiveness ratings, the actual website, and photograph ratings.

Variable	Model 4 (1738 observations, 87 participants)			Model 5 (1738 observations, 87 participants)				Model 6 (1738 observations, 87 participants)			
	Estimate	SE	SD	Estimate		SE	SD	Estimate		SE	SD
Intercept	−0.04	0.09	0.44	−0.05		0.09	0.40	−0.06		0.08	0.39
Attractiveness difference score	0.13^a^	0.05	0.20	0.15^a^		0.05	0.21	−0.03		0.06	0.28
Self-disclosed STI^b^	−0.15	0.11	0.10	−0.14		0.10	0.07	−0.10		0.10	0.09
Intelligence difference score	−0.11^c^	0.05	0.12	—^d^		—	—	—		—	—
Condom use difference score	—	—	—	−0.35^a^		0.07	0.21	—		—	—
Multiple partners difference score	—	—	—	—		—	—	0.38^a^		0.07	0.21

^a^*P*<.01.

^b^STI: sexually transmitted infection.

^c^*P*<.05.

^d^Not entered into the regression.

## Discussion

### Principal Findings

Young adults and adolescents engaging in online dating have to generate quick intuitive judgments when making choices about their interactions with potential sexual partners. Like other decision makers, when they lack statistical estimates, they may rely on heuristics to judge risk [30]. Online dating invites such heuristic judgments in decisions about engaging others as potential sexual partners. In this study, we examined the potential role of an attractiveness heuristic in sexual risk judgment by asking participants to predict which of two photographs came from a website for individuals with self-reported STIs. We assumed that participants would automatically evaluate the target’s attractiveness and then apply the attractiveness heuristic to that judgment.

We did find that attractiveness predicts judgments about STI risk. However, the direction of the association was contrary to our prediction that STIs would seem less likely in more attractive individuals (ie, observing a “halo effect”). Instead, attractiveness appeared to be used as a cue for higher sexual risk. Analyses incorporating other variables led us to an alternative post hoc explanation, which is consistent with research findings that attractive people are perceived as more sexually promiscuous [10,11]. In our study, the relationship between judgments of attractiveness and STI risk appeared to be mediated by judgments of target individuals’ numbers of partners; more attractive individuals are considered to have more partners and hence a greater STI risk. Thus, the archetype of an attractive individual may not be associated with “purity” as much as with “opportunity.” It is also possible that the confidence judgments evoked more analytical and less heuristic thinking, producing more measured inferences about sexual “opportunity,” even in a context designed to encourage snap judgments.

Whatever processes guided their judgments, participants were unable to predict which photographs were drawn from the website with STI disclosure, despite incentives for accuracy. Moreover, they showed overconfidence that is typical of difficult tasks [31]. Their predictions were related to the normatively valid cue of whether the target was rated (by the pretest sample) as someone likely to use condoms and, perhaps, with greater intelligence (Table 3). However, those perceptions appeared to reflect judgments of attractiveness, suggesting sound inferences based on unsound assumptions (Multimedia Appendix 1). As seen in model 6 (Table 3), inferences reflecting attractiveness appear to be subsumed by inferences regarding multiple sexual partners. Given how we created the stimulus set, attractiveness was not a valid cue for predicting a photograph’s origin and yet it played a role in participants’ judgments.

### Limitations

Our study had several notable limitations. First, photograph pretesting was limited to judgments by male and female individuals identifying as heterosexual. Thus, it is possible that the attractiveness ratings for these photographs did not reflect the perceptions of the mixed heterosexual and bisexual participants in our experimental study sample. However, there is evidence that judgments of attractiveness of same and other-sex individuals differ by gender but not sexual orientation [32]. To the best of our knowledge, there is no analogous evidence regarding judgments of STI risk and our sample size did not allow subanalyses by sexual orientation. Understanding how such judgments relate to sexual orientation is a topic for future research, and it is particularly relevant given the popularity of dating apps among gay, bisexual, and other men who have sex with men, as well as their higher risk of contracting STIs [2].

Our convenience sample had a gender imbalance, perhaps reflecting greater interest among male participants (65%) in an experiment about “online dating.” As such, our results are more generalizable to those who are interested in online dating. Within the constraints of our sample, STI predictions were unrelated to gender, gender match with photographs, age, or self-reported STIs (Table 2).

While our sample consisted primarily of college students recruited through a university student pool and other young people recruited in a public setting, we did have a sizable proportion (15/87, 17%) of participants aged ≥25 years. Our results did not differ by age group; however, the lack of age-based exclusion in our recruitment procedures should be noted when interpreting the findings in the pediatric context. Moreover, by advertising the topic, as well as recruiting some participants in person, we may have biased the recruited sample.

According to participants’ self-reports, 41% (36/87) had no sexual encounters and 36% (31/87) had more than 10 encounters. The large representation of sexually inexperienced individuals is unlike much sex-related research [33-35] and higher than reports in national samples of this age group [36]. We cannot predict how this or other selection processes might have influenced our results. We attempted to focus participants on the images, rather than the individuals in them, by presenting targets who participants would never meet and by providing financial incentives for accuracy. This approach deprived participants of both valid and invalid cues available in everyday life, the most important of which may be the website on which the photograph appeared. Thus, our results suggest a form of heuristic thinking that young people may use, without indicating its power or prevalence.

### Conclusions

Romantic interest in a target has been found to be driven primarily by sexual attractiveness [35]. For many people, the attractiveness of a potential partner’s online image affects whether to proceed in engaging with them [37]. Using an experimental task that involved rapid judgments of photographs, we found that judgments of STI risk were related to both relevant risk factors and the irrelevant cue of attractiveness. The role of attractiveness appears to have been mediated by the rapid inference that attractive people have more sexual partners and hence greater STI risk. Although participants predicted STI risk at chance levels (47%), they had moderate confidence in their predictive ability (mean confidence 67%). Reliance on heuristic judgments of risk from visual cues alone, paired with a misplaced confidence in the ability to identify risk among others, could contribute to the higher rates of STIs among those who use online dating sites to initiate sexual encounters [38-40].

These findings may help inform conversations between health care providers and their young patients, providing content for STI counseling that many are eager to provide [41]. Such conversations offer one way to address the medical community’s call for more proactive approaches to stem the rise in STI rates [42]. Those conversations may include discussions on the validity of the cues available in online dating apps. This research is a formative step toward understanding young people’s inference processes when using apps that play central roles in many of their lives. Further research should examine heuristics that might better inform the snap judgments of sexual risk that accompany “swiping left or right.”

## Appendix

Multimedia Appendix 1 Descriptive statistics and Spearman rank correlations for pretest judgments of target photographs.

## Abbreviations

IRB: institutional review board
STI: sexually transmitted infection
