# Supplementary material for: Inferring Sexually Transmitted Infection Risk From Attractiveness in Online Dating Among Adolescents and Young Adults: Exploratory Study
Source: J Med Internet Res. 2020 Jun 9;22(6):e14242. doi: 10.2196/14242 (PMC7313732; doi:10.2196/14242)
Supplement: Multimedia Appendix 1 [file jmir_v22i6e14242_app1.docx]

Multimedia Appendix 1. Descriptive statistics and Spearman rank correlations for pretest judgments of target photographs.

|  | **M**  **(SD)** | **Attractiveness** | **Intelligence** | **Frequency of Condom-Use** | **Multiple Sexual Partners** | **Decision**  **Competence** |
| --- | --- | --- | --- | --- | --- | --- |
| **Attractiveness** | 3.36 (1.61) |  | .25 | .10 | .20 | .21 |
| **Intelligence** | 4.05 (1.39) |  |  | .44 | -.26 | .67 |
| **Frequency of Condom-Use** | 4.69 (1.55) |  |  |  | -.39 | .50 |
| **Multiple Sexual Partners** | 3.72 (1.63) |  |  |  |  | -.33 |
| **Decision Competence** | 4.11  (1.45) |  |  |  |  |  |
